# Advanced AI techniques for classifying Alzheimer’s disease and mild cognitive impairment

**DOI:** 10.3389/fnagi.2024.1488050

**Published:** 2024-11-29

**Authors:** Sophie Tascedda, Pierfrancesco Sarti, Veronica Rivi, Claudia Savia Guerrera, Giuseppe Alessio Platania, Mario Santagati, Filippo Caraci, Johanna M. C. Blom

**Affiliations:** ^1^Plateforme de Bioinformatique, Centre Hospitalier Universitaire Vaudois (CHUV), Lausanne, Switzerland; ^2^Service de Chimie Clinique CHUV, Lausanne, Switzerland; ^3^Faculté de Biologie et de Médecine, Université de Lausanne, Lausanne, Switzerland; ^4^Department of Biomedical, Metabolic and Neural Sciences, University of Modena and Reggio Emilia, Modena, Italy; ^5^Department of Adult Psychiatry and Psychotherapy, Psychiatric Hospital, University of Zurich, Zurich, Switzerland; ^6^Department of Educational Sciences, University of Catania, Catania, Italy; ^7^ASP3 Catania, Department of Mental Health, Alzheimer Psychogeriatric Centre, Catania, Italy; ^8^Department of Drug and Health Sciences, University of Catania, Catania, Italy; ^9^Unit of Neuropharmacology and Translation Neurosciences, Oasi Research Institute – IRCCS, Troina, Italy; ^10^Centre for Neuroscience and Neurotechnology, University of Modena and Reggio Emilia, Modena, Italy

**Keywords:** artificial intelligence, graph convolutional networks, machine learning, deep learning, dementia, neural networks

## Abstract

**Background:**

Alzheimer’s disease and mild cognitive impairment are often difficult to differentiate due to their progressive nature and overlapping symptoms. The lack of reliable biomarkers further complicates early diagnosis. As the global population ages, the incidence of cognitive disorders increases, making the need for accurate diagnosis critical. Timely and precise diagnosis is essential for the effective treatment and intervention of these conditions. However, existing diagnostic methods frequently lead to a significant rate of misdiagnosis. This issue underscores the necessity for improved diagnostic techniques to better identify cognitive disorders in the aging population.

**Methods:**

We used Graph Neural Networks, Multi-Layer Perceptrons, and Graph Attention Networks. GNNs map patient data into a graph structure, with nodes representing patients and edges shared clinical features, capturing key relationships. MLPs and GATs are used to analyse discrete data points for tasks such as classification and regression. Each model was evaluated on accuracy, precision, and recall.

**Results:**

The AI models provide an objective basis for comparing patient data with reference populations. This approach enhances the ability to accurately distinguish between AD and MCI, offering more precise risk stratification and aiding in the development of personalized treatment strategies.

**Conclusion:**

The incorporation of AI methodologies such as GNNs and MLPs into clinical settings holds promise for enhancing the diagnosis and management of Alzheimer’s disease and mild cognitive impairment. By deploying these advanced computational techniques, clinicians could see a reduction in diagnostic errors, facilitating earlier, more precise interventions, and likely to lead to significantly improved outcomes for patients.

## 1 Introduction

In clinical neurology and geriatrics, diagnosing and treating neurodegenerative conditions such as Alzheimer’s disease (AD) and mild cognitive impairment (MCI) is particularly challenging due to the physical and cognitive vulnerabilities of elderly patients. These challenges are exacerbated by the diseases’ complexity, their phenotypic similarities, and frequent comorbidities, often leaving patients in a diagnostic limbo without access to specific therapies (Wen et al., 2020). Differentiating between MCI, considered a transitional phase between normal cognitive aging and dementia, and mild AD is especially difficult due to their overlapping symptoms (Sperling, 2011; Jack et al., 2018).

Several diagnostic criteria, such as prodromal AD and MCI due to AD, have been proposed based on biomarkers that reflect brain changes typical of AD. These biomarkers include episodic memory decline, hippocampal atrophy on MRI, abnormal cerebrospinal fluid (CSF) biomarkers (e.g., low amyloid-β42, increased tau), and abnormal PET scan results showing amyloid and tau deposits or reduced glucose metabolism in temporoparietal regions (Armananzas et al., 2013; Sarraf et al., 2016; Schirrmeister et al., 2017; Leandrou et al., 2018). While these biomarkers are used in specialised centres, their integration into routine clinical practice has been slow (Albert et al., 2011; McKhann et al., 2011; Dubois, 2014). There are still uncertainties about the benefits and potential drawbacks of diagnosing prodromal AD or MCI due to AD, particularly regarding the emotional impact on patients and the unpredictability of disease progression (Khan et al., 2020). Predicting the progression from MCI to dementia with accuracy remains a challenge (Ding et al., 2023; Korolev et al., 2016). Traditionally, diagnosing AD relied on clinical assessment and cognitive testing, such as the Mini-Mental State Examination (MMSE) and Montreal Cognitive Assessment (MoCA) (Li et al., 2015; Wang et al., 2022). Advances in technology have introduced tools like MRI, PET, DTI, biomarkers, and CSF analysis to detect AD more objectively (Sørensen et al., 2016; Wang et al., 2018; Li, 2020; Rodríguez-Santiago et al., 2024). New criteria for diagnosing and staging Alzheimer’s disease have recently been proposed that also consider advances in biological markers and brain analysis techniques (Jack et al., 2024). However, these methods have limitations: clinical assessments can be subjective, and early-stage AD may not be detectable with sufficient sensitivity using brain imaging techniques like MRI (Bron et al., 2015; Rathore et al., 2017; Parisot, 2018; Spasov et al., 2019; Zhao and He, 2019).

Recently, machine learning (ML) models have been applied to AD research to identify patterns associated with the disease, which may allow for earlier interventions and the possibility of slowing disease progression (Ju et al., 2017; Wang et al., 2018; Lian et al., 2020; Li, 2020). Beyond AD and MCI, Artificial Intelligence (AI) applications are beginning to revolutionize the diagnosis and management of other types of dementia and neurodegenerative disorders. For example, deep learning models and convolutional neural networks (CNNs) have shown potential in distinguishing Parkinson’s Disease Dementia and Lewy Body Dementia (Faragó et al., 2023; Altham et al., 2024). Additionally, natural language processing (NLP) tools are emerging as promising methods for early detection of language impairments in Frontotemporal Dementia (Panahi et al., 2024; Vonk et al., 2024), allowing for more accurate differentiation from other forms of dementia and psychiatric conditions. In cases of Vascular Dementia, machine learning models applied to MRI and CT scan analysis have improved diagnostic accuracy by identifying brain changes specific to ischemic processes (Castellazzi et al., 2020). Even in conditions such as Amyotrophic Lateral Sclerosis and Primary Progressive Aphasia, where cognitive symptoms are secondary, AI-based image analysis has proven useful for tracking disease progression (Rezaii et al., 2024). These AI-driven approaches not only enhance diagnostic precision but also support personalized intervention strategies, thereby improving clinical practice and care for diverse types of neurodegenerative patients.

Despite their effectiveness, ML models often lack transparency in real-world healthcare settings, limiting their clinical acceptance. In this complex scenario, AI and advanced imaging techniques are emerging as promising tools to improve AD diagnosis and prediction (Pinto-Coelho, 2023). AI can analyse complex patterns in biomarkers, imaging, and clinical data, potentially enabling more precise and early detection of AD. Such approaches that seek to combine machine learning techniques and medical research are already in place to enhance the discovery of new drug targets and provide more accurate risk stratifications; especially in degenerative diseases (Geraci et al., 2024).

In this study, we explore the potential of AI to enhance the diagnosis and understanding of AD and MCI. We specifically focus on the use of Multi-Layer Perceptron (MLP), Graph Convolution Network (GCN), and Graph Attention Network (GAT) models to analyse and interpret clinical data (Nia et al., 2023).

Our primary objective is to accurately classify patients into three categories: control group, MCI group, or AD group. For this purpose, we applied these three classification models (MLP, GCN, GAT) to a population of patients with MCI and AD, along with a control group. This allowed us to evaluate the performance of these models in correctly classifying patients and healthy subjects, integrating data from demographic variables, neuropsychological tests, and treatment and rehabilitation indicators.

To our knowledge, there are still few studies using AI algorithms in Cognitive Decline classification based on psychological variables, representing a critical area for further research.

## 2 Methods

### 2.1 Participants

The study sample consisted of 214 adults, divided into three groups: the amnesic Mild Cognitive Impairment group with 77 participants (54 females and 23 males, mean age 75.53 ± 7.3), the probable mild Alzheimer’s Disease group with 30 participants (19 females and 11 males, mean age 76.33 ± 6.4), and a Control group comprising 107 participants (78 females and 29 males, mean age 74.06 ± 6.8). The MCI and mild AD groups were recruited from a specialised cognitive impairment service, the “U.O.S. Centro Alzheimer e Psicogeriatria” at ASP3 in Catania, Italy. Specific inclusion and exclusion criteria were applied according to the protocols of the National Institute of Aging (NIA) and the Alzheimer’s Association Work Group for amnesic MCI and AD.

The criteria for MCI due to AD included cognitive concerns reported by the patient, an informant, or a clinician; objective evidence of impairment in one or more cognitive domains, typically memory; preservation of independence in functional abilities; and the absence of dementia. For probable AD, the criteria were cognitive or behavioural symptoms that interfere with daily activities and represent a decline from previous functioning levels, cognitive impairment confirmed through patient history and objective assessment, impairment in at least two cognitive or behavioural domains, insidious onset, clear evidence of worsening cognition, and specific cognitive deficits (either amnestic or non-amnestic). Patients with cognitive impairment due to cerebrovascular disease, dementia with Lewy bodies, or other neurological or non-neurological conditions that could affect cognitive function were excluded. Participants with an age-and education-adjusted MMSE score between 18 and 28 were included, while those with a recent history of cerebral ischemia or psychotic episodes were excluded. Consequently, 107 amnesic MCI and probable mild AD subjects were included in the study. The term “probable” AD is used here because the diagnosis was based solely on clinical signs and symptoms, without genetic or biomarker analyses. The Control group consisted of 107 healthy volunteers with an MMSE score of 28 or higher. Their clinical history, cognitive performance, and daily functioning were assessed to confirm their healthy status and exclude mild neurocognitive disorder, in line with DSM-5-TR and ICD-11 guidelines.

Patients were assessed during their scheduled appointments using the Italian standardised version of the Mini-Mental State Examination, which is recommended by the Italian AIFA (Agenzia Italiana del Farmaco) guidelines for staging cognitive deterioration. The MMSE was initially used to screen participants and divide them into the Control, MCI, and mild AD groups. This division was confirmed by clinical history and performance on other neuropsychological tests, consistent with the aforementioned guidelines.

Information about participants’ usual autonomy was gathered from both the participant and a knowledgeable informant. Additionally, MoCA score above 26 was considered as further assurance of the absence of preclinical cognitive decline, as this threshold was more conservative than other cut-offs proposed for the Italian population in distinguishing healthy individuals from those with MCI.

All participants provided informed consent for the processing of their data and for their publication and were individually tested in a single session by clinical psychologists experienced in dementia. After the screening process, the study sample included more women than men, reflecting the sex prevalence of cognitive impairment in both Italy and globally.

### 2.2 Data preparation and representation

To ensure the networks would receive clean and standardised input, a series of processing steps was performed. Numerical features such as Age, Education, Mini-Mental State Examination, Montreal Cognitive Assessment (MoCA), Frontal Assessment Battery (FAB), and Hamilton Depression Rating Scale (HDRS) scores were standardised to achieve zero mean and unit variance. Categorical features, including Sex, Comorbidity, Treatment, and Rehabilitation, were one-hot encoded to facilitate their incorporation into the models. This preprocessing step scaled numerical features and encoded categorical ones, ensuring all input data was uniformly formatted for the models.

**Figure 1:**
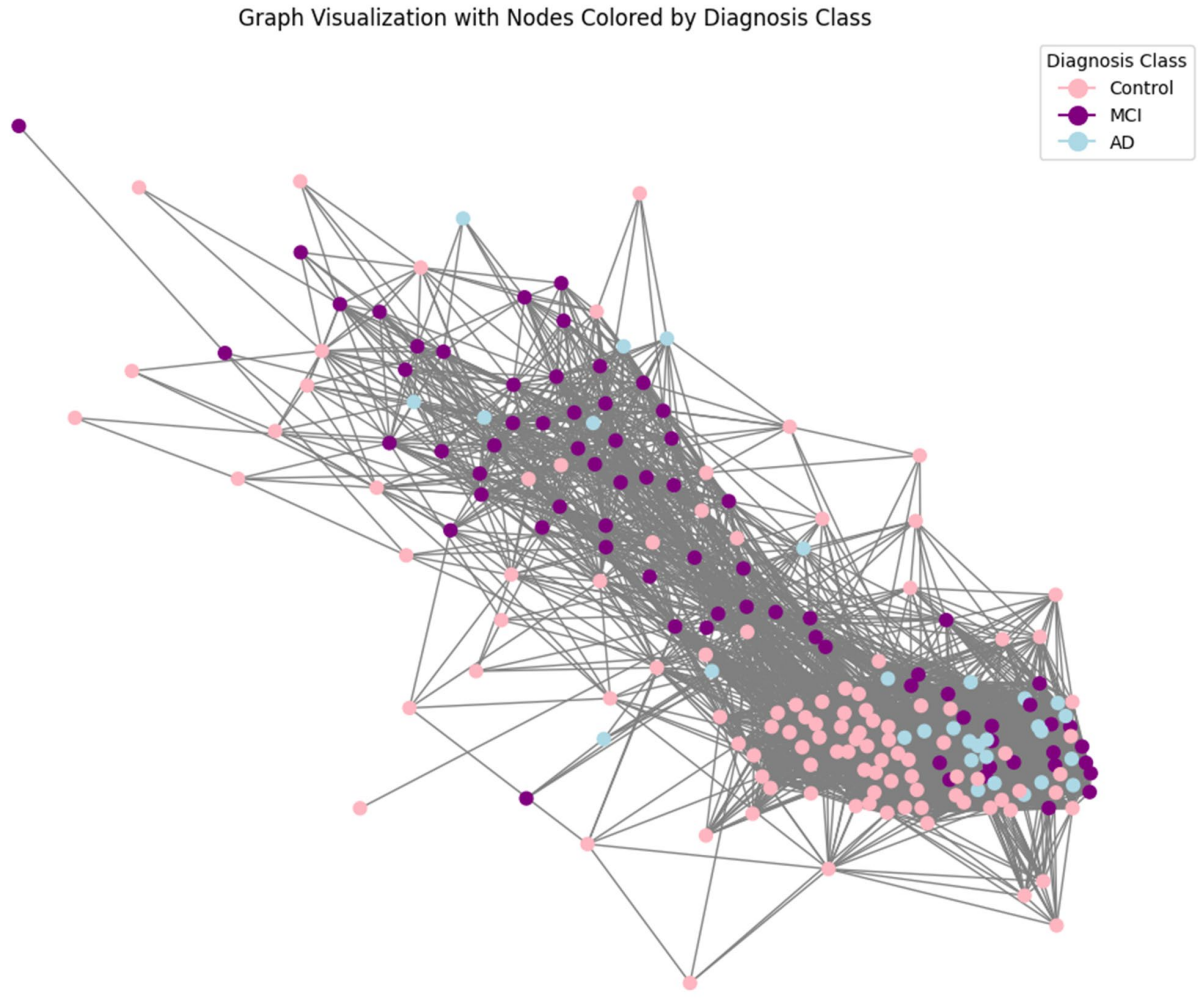
Graph representation of patient data. This figure illustrates the graph-based representation of the data, where each node represents a patient, and edges represent the similarity between patients based on their features. The similarity between two patients (nodes) was computed using a Gaussian Radial Basis Function (RBF) kernel applied to the Euclidean distance between their feature vectors. This transformation ensures that patients with similar clinical profiles are more strongly connected. Nodes are linked by edges when their similarity exceeds a predefined threshold (here set to keep top 20% similarities), resulting in a graph structure that preserves meaningful relationships critical for classification tasks.

A crucial aspect of our approach is the representation of patient data as a graph to leverage the relational information among patients. This graph-based approach allows us to model the complex interactions between different patients based on their clinical features, facilitating more nuanced and accurate predictions (Parisot, 2018). We first computed a distance matrix using the Euclidean distance between each pair of patients based on their scaled features. The Euclidean distance is a commonly used metric that measures the straight-line distance between points in multi-dimensional space, effectively capturing differences in clinical profiles. Next, we transformed it into a similarity matrix using a Gaussian (RBF) kernel, which is particularly suitable for converting distances into similarities because it ensures that patients with similar clinical features (i.e., closer in the feature space) have higher similarity scores. Since connecting every pair of patients would make the graph too dense and potentially noisy, to create edges between nodes (patients) in the graph we needed a way to select only the most meaningful connections. We applied a percentile-based thresholding method: instead of choosing a fixed similarity value as the cutoff, we calculated the 80th percentile of similarity scores in the matrix. This means we only kept the top 20% of similarity scores to define edges, connecting each patient only with those with the highest similarity. This approach allowed us to retain the most significant connections while discarding weaker, less relevant ones, resulting in a sparse graph. After thresholding, we generated an edge index for the graph, representing pairs of patients (nodes) with similarity scores above this threshold. This index list is essential for feeding the graph structure into GNN models. The edge index acts as a roadmap for the GNN, guiding it in learning from the structured connections between patients. The visualization of the obtained graph is reported in Figure 1. The resulting graph has 214 nodes (representing individual patients) and 4,472 edges, giving an average degree of 20.9 connections per patient. The graph is undirected, meaning connections are mutual, with no self-loops, and contains 9 isolated nodes (patients with no connections to others due to dissimilarity), which are omitted from the visual representation for clarity.

Finally, we divided the data into training, validation, and test sets. Given the class imbalance (77 MCI, 30 AD, and 107 Control), the split was performed with class awareness to ensure proportional representation of each category. Additionally, the dataset has a gender imbalance, with 151 females (70.56%) and 63 males (29.44%). We confirmed that these proportions were maintained across the subsets: the training set consists of 71.43% females and 28.57% males, the validation set has 76.67% females and 23.33% males, and the test set includes 66.15% females and 33.85% males.

### 2.3 Model selection and architecture

The classification was carried out using 3 different models: MLP, GCN, and GAT. Each model was chosen for its specific strengths and suitability for the task and the data. The models and architectures are explained extensively below, and a summary of their characteristics has been included in Table 1.

**Table 1:** Summary table of the three models used with their characteristics and architectures.

Feature	MLP	GCN	GAT
Input features	Node Features (11)	Node Features (11)	Node Features (11)
Exploits graph structure	No	Yes (nodes & edges)	Yes (nodes & edges)
Hidden layers	2 Dense layers	2 Graph convolutional layers	2 Graph attention layers
Neurons/units per layer	16, 8	16 (layer 1)	16 per attention head (layer 1), 8 attention heads
Activation function	ReLU	ReLU	ELU
Dropout rate	0.1	0.1	0.2
Output layer	3 Neurons	3 units (final convolutional layer)	3 units (final attention layer)
Additional features	–	Graph structure aggregation	Graph structure aggregation & attention mechanism

The MLP is a type of feedforward artificial neural network that consists of multiple layers of nodes, or neurons, each of which uses a nonlinear activation function such as ReLU (Rectified Linear Unit). The MLP was fed the node features extracted from the graph, capturing complex nonlinear relationships within the data. Although MLPs do not inherently exploit the graph structure, they provide a robust baseline for comparison with graph-based models by focusing on individual node features.

In this study, an input layer receives the node features extracted from the graph, consisting of 11 input features per node. Then two hidden layers with 16 and 8 neurons, respectively. The ReLU activation function introduces non-linearity, allowing the model to learn complex patterns in the data. A dropout rate of 0.1 is used to prevent overfitting by randomly dropping neurons during training. The final output layer consists of 3 neurons, corresponding to the three classification classes (control, MCI, Alzheimer’s). No activation function is applied at this layer, as it is followed by the CrossEntropyLoss (Mao et al., 2023) during training.

GCNs are specialised neural networks designed to operate directly on graph structures. They apply convolutional operations on the graph, allowing the model to capture the intricate interactions between nodes (patients) and edges (shared clinical features). GCNs work by combining information from neighbouring nodes, helping the model learn patterns that traditional models might miss. This makes them particularly effective for tasks where relational information is key to understanding the data’s complexities. In clinical settings, GCNs have been used for various applications, such as predicting and interpreting cancer survival outcomes (Ramirez et al., 2021) and revealing network-level functional dysconnectivity in conditions like schizophrenia (Lei et al., 2022).

As in the previous method, the input layer processes the node features extracted from the graph. The first convolution layer applies a graph convolution operation to the input features, outputting 16 hidden units per node. The ReLU activation function introduces non-linearity, enabling the model to learn from the graph’s structure and a dropout rate of 0.1 is used to prevent overfitting. The second convolution layer further processes the output from the first layer, reducing the dimensionality to 3 output units per node, corresponding to the classification labels. After aggregation, the output layer directly maps these features to class probabilities. The model applies a SoftMax function (implicitly through loss functions like cross-entropy loss) to convert the raw scores into probabilities for each class.

GATs are an extension of GCNs that incorporate attention mechanisms. Attention mechanisms enable the model to dynamically weigh the importance of different edges in the graph, allowing it to focus more on the most relevant connections while potentially ignoring less important ones and leading to a deeper understanding of how different patients are connected (Vaswani et al., 2023). In healthcare, GATs enhance diagnostic predictions by leveraging relational data. For example, they have been tested for diagnosing autism spectrum disorder by analysing brain networks to identify key connections (Yang et al., 2021), or applied to COVID-19 diagnosis task, using chest X-ray images to highlight critical regions indicative of the disease.

The input layer processes the node features, like the other models. The first attention layer uses a multi-head attention mechanism with 8 attention heads, each outputting 16 hidden units. The ELU activation function is employed to introduce non-linearity and is here chosen instead of ReLU as it provides better gradient flow and faster convergence in deep networks with attention mechanisms. This layer computes attention coefficients for each edge, allowing the model to focus on the most relevant connections. The second attention layer processes the outputs from the first and produces final scores for each class, which are used to compute probabilities via a SoftMax function in the loss layer (like for GCN). For the GAT a higher dropout rate of 0.2 compensates for the increased model complexity due to attention mechanisms, reducing the risk of overfitting and enhancing model generalisation.

Each network consists of an input layer, hidden layers, and an output layer, tailored to capture different aspects of the data for effective classification of control, MCI, and Alzheimer’s patients. The MLP serves as a baseline model, focusing solely on node features to capture non-linear relationships. In contrast, GCN and GAT exploit both node and edge information, leveraging the graph structure to understand interactions between patients.

### 2.4 Training and evaluation-

The training process for each model involved:

1. Hyperparameter tuning: the goal was to find the optimal combination of parameters that maximise model performance.
  - Parameters Tuned: we experimented with various combinations of learning rates and weight decays. Learning rates control how much the model’s weights are adjusted during training, while weight decay helps prevent overfitting by adding a regularisation term.
  - Approach: each model was trained for 100 epochs using a grid search approach to explore different hyperparameter settings. This systematic exploration allowed us to identify the most effective configuration for each model.
2. Train/validation split: To evaluate model performance and prevent overfitting, we split the training data into a training set and a validation set. This split ensures that the model is tested on unseen data during training. The validation set was used to track key performance metrics such as accuracy, positive predictive value (PPV) (Safari et al., 2015), recall, and loss throughout the training process. This monitoring enabled early detection of overfitting and informed decisions about model adjustments. Accuracy provides a general measure of the model’s overall correctness across all classes. Precision PPV, commonly referred to as precision in machine learning literature, is used in clinical practice to understand how useful and reliable a diagnostic test is in everyday practice, particularly in correctly identifying patients who actually have a disease or condition, as it indicates the proportion of positive identifications that were actually correct. It reflects the proportion of positive identifications that were actually correct, crucial for minimising false positives in clinical diagnoses, and Recall (or sensitivity) indicates the proportion of actual positives that were correctly identified, ensuring that true cases of the disease are not missed. To provide a balanced assessment across all classes, we used weighted recall in our analysis.
3. Training process:
  - Optimizer: We used the Adam optimizer (Kingma and Ba, 2017), a popular choice for training neural networks due to its adaptive learning rate and ability to handle sparse gradients.
  - Loss function: cross-entropy loss was employed for the classification task. This loss function is well-suited for multi-class classification problems, as it calculates the difference between predicted and true class probabilities.
  - Epochs: Each model was trained for 100 epochs, allowing sufficient time for learning while preventing excessive training that could lead to overfitting.
  - Recording metrics: Throughout the training process, we recorded training losses for each epoch to monitor the model’s learning progress. This information was used to plot training curves and assess convergence.
  - Validation checks: After each epoch, the model’s performance on the validation set was evaluated. This step was crucial for determining whether the model’s performance was improving.
4. Post-Training evaluation: After training, the best-performing model for each architecture was selected based on validation metrics, including accuracy, precision, and recall.
  - Testing: The final evaluation was performed on the test set, using the model configuration that achieved the best validation results. This test ensures that the model’s performance is robust and generalises well to new, unseen data.

All analyses and model implementations were built and executed using Python 3.10.12.

## 3 Results

In our study, we evaluated the performance of three models—MLP, GCN and GAT—with the primary goal of accurately classifying patients into control, MCI, or AD categories. The evaluation was based on key metrics that are highly relevant in a clinical setting: accuracy, PPV, and recall. The resulting models are reported below:

### 3.1 MLP

**Figure 2:**
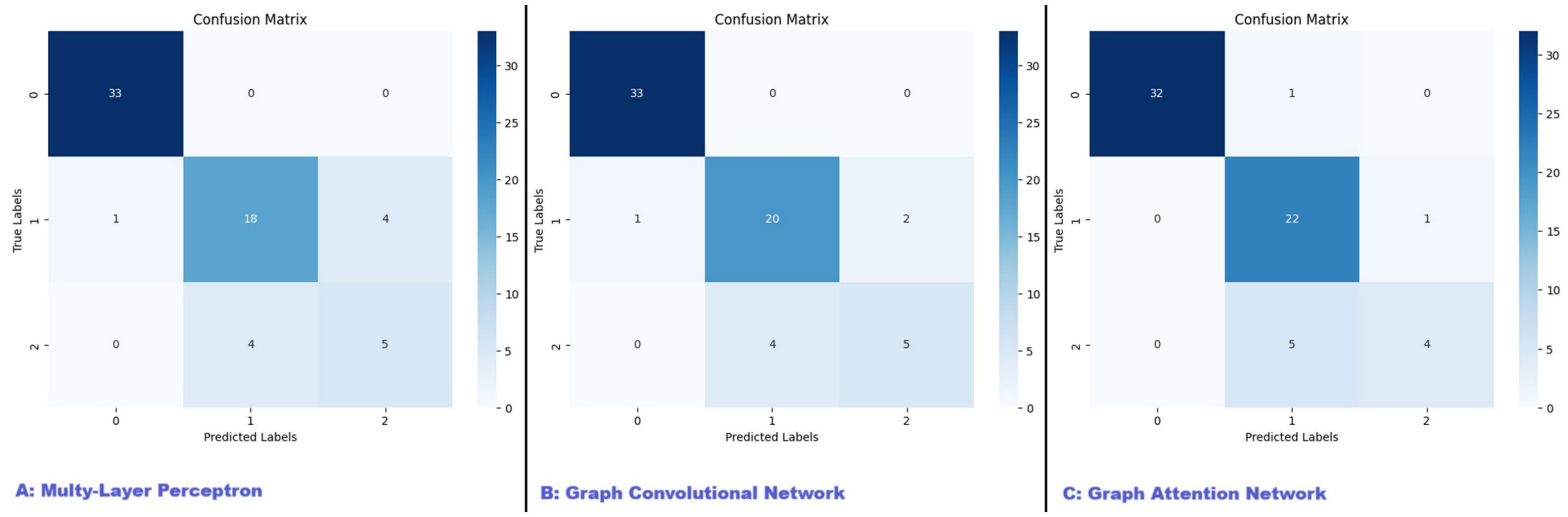
Confusion matrices for MLP, GCN, and GAT models. This figure presents the confusion matrices for the three models evaluated in our study: **(A)** Multi-Layer Perceptron (MLP), **(B)** Graph Convolutional Network (GCN), and **(C)** Graph Attention Network (GAT). The confusion matrices illustrate the performance of each model in classifying patients into control, mild cognitive impairment (MCI), and Alzheimer’s disease (AD) categories. The diagonal elements represent the number of correctly classified instances for each class, while the off-diagonal elements indicate misclassifications.

The MLP model was trained with the best performing parameters being a learning rate of 0.01 and a weight decay of 0.0001. The model achieved an overall test accuracy of 86.15%. The weighted PPV and recall were 85.92 and 86.15%, respectively. The classification performance across different categories, for which the confusion matrix is reported in Figure 2A, was the following:

- **Control**: PPV of 97%, Recall of 100%.
- **MCI**: PPV of 82%, Recall of 78%.
- **Alzheimer’s**: PPV of 56%, Recall of 56%.

While the MLP showed high performance in the control group, its recall for the AD’s group, with only 56% of true cases being correctly identified, was less performant. This limitation suggests that while the MLP is capable of capturing nonlinear relationships within the data, its lack of integration with the graph structure limits its effectiveness in fully exploiting the relational information inherent in the patient data. The model’s classification report highlights this, showing a high precision but a relatively low recall for the Alzheimer’s class, which could lead to missed diagnoses in a real-world setting.

### 3.2 GCN

The GCN model was trained with optimal hyperparameters being a learning rate of 0.01 and a weight decay of 0.0005. The model reached an overall accuracy of 89.23%, with a weighted PPV of 88.65% and a weighted recall of 89.23%. The per-class results, reported visually with the confusion matrix in Figure 2B, were as follows:

- **Control**: PPV of 97%, Recall of 100%.
- **MCI**: PPV of 83%, Recall of 87%.
- **Alzheimer’s**: PPV of 71%, Recall of 56%.

The GCN demonstrated a more balanced performance across all classes, particularly improving the recall for Alzheimer’s patients to 71%. This improvement indicates that the GCN effectively captures the underlying structure and interactions between patients, which are critical in the context of complex clinical data. The per-class values were:

### 3.3 GAT

The GAT model further extended the capabilities of the GCN by incorporating attention mechanisms, allowing the model to dynamically weigh the importance of different edges within the graph. The model was trained with the best-performing hyperparameters being a learning rate of 0.01 and weight decay of 0.0005. The GAT achieved the highest test PPV at 89.65% and matched the GCN’s weighted recall of 89.23%, with an overall accuracy of 89.23%. The per-class performance, also reported in the form of confusion matrix in Figure 2C, was the following:

- **Control**: PPV of 100%, Recall of 97%.
- **MCI**: PPV of 79%, Recall of 96%.
- **Alzheimer’s**: PPV of 80%, Recall of 44%.

While the GAT model excelled in precision, particularly in the Alzheimer’s group, its recall in this category was lower, indicating that the model may prioritize precision over recall, which could be a concern in clinical settings where identifying all cases is critical.

## 4 Discussion

Differentiating between MCI and AD remains a challenging task in clinical practice. MCI, often considered a transitional phase between normal cognitive aging and dementia, shares many overlapping symptoms with early-stage AD, making the two conditions difficult to distinguish (He, 2016; Lee, 2023). Traditional diagnostic methods are generally effective for detecting advanced AD (Dubois, 2014; Jack et al., 2018; McKhann et al., 2011), but they struggle in early stages, where symptoms are subtle and biomarkers may not yet reveal clear pathological distinctions (Sperling, 2011; Parisot, 2018). Early detection is essential to enable timely interventions, as this could improve outcomes for individuals in the MCI phase by delaying or even preventing progression to AD.

To address these challenges, we employed advanced AI models—MLP, GCN, and GAT—to classify patients into control, MCI, and AD categories (Albert et al., 2011; Kipf and Welling, 2017). This study is among the first to apply these sophisticated models specifically to differentiate MCI from AD, shedding light on unique diagnostic challenges associated with each condition. Our findings highlight the potential of AI for early diagnosis, especially in capturing subtle cognitive changes indicative of MCI or AD, and emphasize AI’s role in tackling complexities that traditional approaches might miss (Bertozzi, 2019; Zhang et al., 2019; Jiang, 2020; Kumar and Min, 2020; Veličković, 2018; Yao, 2020).

Each model demonstrated strengths and limitations in achieving accurate classifications. All models performed well in distinguishing control patients, with both precision and recall rates approaching 100%. However, separating MCI from AD proved significantly more challenging, particularly for the AD group, which displayed consistently lower recall rates across all models. The GCN model exhibited a more balanced performance, improving recall for AD to 71% compared to MLP’s recall of 56%. This suggests that GCN’s capacity to leverage the graph structure, capturing patient relationships, offers an advantage in neurodegenerative contexts where such interdependencies may reflect disease dynamics. The GAT model achieved the highest overall precision but at the cost of a lower recall rate for AD (44%), indicating a trade-off: while GAT effectively reduces false positives, it may miss true AD cases—a critical consideration in clinical settings where missed diagnoses could have serious consequences.

All three methods used perform optimally in distinguishing the control group from the other two groups. However, they show higher accuracy in classifying MCI compared to AD. This discrepancy can be attributed to two main factors.

First, the choice of neuropsychological tests plays a crucial role. Assessments such as the MoCA and MMSE are particularly effective in detecting mild cognitive decline, which is characteristic of MCI. Designed to identify early cognitive impairments, these tests are sensitive to the initial stages of decline, providing a clearer distinction from AD as symptoms become more pronounced. Additionally, the Beck Depression Scale and Apathy Evaluation Scale further aid differentiation: MCI patients often display more depressive symptoms, while apathy is more common in AD. Second, the smaller sample size of the AD group likely influenced the algorithms’ learning processes, potentially limiting accuracy during testing and validation. Increasing the sample size could enhance the model’s capacity to differentiate AD from MCI and improve generalizability.

The accuracy levels achieved in our study are consistent with similar research relying solely on neuropsychological data for AD and MCI diagnosis. For example, the Chinese Neuropsychological Consensus Battery achieved approximately 80% accuracy in identifying individuals at risk based on neuropsychological data alone (Gu et al., 2023). Additionally, studies employing natural language processing to analyse semantic variations and predict progression from MCI to AD report comparable accuracy (Amini et al., 2024), underscoring the value of neuropsychological and language-based data in early diagnosis. Studies that incorporate additional data types, such as MRI or PET imaging through CNNs and hybrid models, report even higher accuracy, underscoring the added value of detailed structural and functional brain data (Arya et al., 2023; Baskar et al., 2023). However, in clinical practice, neuroimaging is often reserved as a confirmatory tool due to its cost and limited accessibility. Consequently, neuropsychological assessments remain primary tools in initial diagnostic impressions, and AI models developed exclusively on such data hold practical relevance for clinical use.

Our findings highlight the complexity of accurately diagnosing AD using AI models, particularly when distinguishing it from MCI (Wang, 2020; Amin, 2021; Hsu, 2021; Liu, 2021; Ma, 2021; Zhang, 2021). The lower recall rates for AD suggest that, despite advancements in AI, the subtle and overlapping symptoms of AD and MCI remain a significant challenge. This complexity underscores the necessity of continuous refinement in AI algorithms and the integration of more diverse and comprehensive data sets.

The application of AI in neurological diagnostics represents a promising advancement, potentially improving diagnostic accuracy and consistency, especially in cases where traditional methods might fall short. Yet, the challenges encountered in our study emphasize that AI models, while valuable, must still be complemented by clinical expertise. Our approach using GNN and GAT is well-suited for clinical scenarios where neuropsychological and demographic data are primary indicators. Such methodology could be implemented in primary care settings or memory clinics, where access to advanced diagnostic tools like MRI or PET scans is limited. By leveraging GNNs and GATs, clinicians can examine relationships among patients with similar clinical characteristics, supporting more tailored diagnostic pathways and treatment plans that optimize available resources.

Looking ahead, research should prioritize refining AI algorithms by integrating comprehensive datasets and exploring hybrid methodologies. Achieving a balance between precision and recall is critical to reduce false negatives, which can be particularly costly in early-stage diagnoses. Integrating diverse data sources will deepen our understanding of symptom networks, ultimately leading to more effective tools for early and accurate diagnosis. With continued improvements, AI has the potential to transform the approach to neurodegenerative disease diagnosis, marking a shift toward more proactive and personalized care in neurology.

## Data Availability

The raw data supporting the conclusions of this article will be made available by the authors, without undue reservation.
